# Pheochromocytoma induced cardiomyopathy in a young man: a case report

**DOI:** 10.1093/omcr/omaa128

**Published:** 2021-01-23

**Authors:** Christopher Ryan Zörner, Ulrik Dixen, Birgitte Grønkær Toft, Mie Skjøttgaard Ynddal, Peter Sommer, Jens Dahlgaard Hove, Martin Steen Frydland

**Affiliations:** 1 Department of Cardiology, Hvidovre Hospital, Copenhagen, Denmark; 2 Department of Pathology, Rigshospitalet, Copenhagen, Denmark; 3 Department of Urology, Rigshospitalet, Copenhagen, Denmark

## Abstract

Pheochromocytoma is a tumor arising from the adrenal medulla, most frequent benign and, due to the excretion of catecholamines, a rare cause of hypertension. The diagnosis of pheochromocytoma can be challenging because of its episodic nature, unspecific symptoms and rarity. Consequently, treatment can be delayed with serious consequences for the patient. We present a case report regarding a young man with episodes of severe hypertension over a period of at least 9 years. Ultimately, with a possible trigger effect from the intake of multiple energy drinks, the patient presented with severe hypertension, symptoms mimicking acute coronary syndrome, abnormal laboratory parameters and echocardiography suggestive of severe cardiomyopathy. The patient’s pheochromocytoma was incidentally identified in a computed tomography scan during the initial workup. Although a rare condition, pheochromocytoma should be considered as a differential diagnosis, especially in young patients presenting with unexplained hypertension, chest pain and cardiac dysfunction.

## INTRODUCTION

Pheochromocytoma is a rare catecholamine producing tumor that can go undetected over a long period of time, until it presents with a myriad of symptoms, often imitating other diseases and disorders. We present a case report regarding a young man with episodes of severe hypertension over a period of at least 9 years. Ultimately, with a possible trigger from the intake of multiple energy drinks, he presents with severe hypertension and symptoms mimicking acute coronary syndrome.

## CASE REPORT

A 33-year-old man, without known co-morbidities, was brought into the emergency department with sudden onset of headache and severe central chest pain, radiating to the back. The patient arrived 3.5 hours after the onset of symptoms and was still in severe pain.

The blood pressure was 180/125 mmHg, heart rate 105 beats per minute (bpm), temperature 36.1°C, oxygen-saturation 98% and the respiratory-rate was 28 per minute. An arterial blood gas (ABG) revealed significant lactate acidosis with pH 7.14, pO2 of 15.4 kPa, pCO2 of 4.5 kPa, bicarbonate of 12 mEq/L, base excess of −16 mmol/L and lactate value of 12 mmol/L. A 12 lead electrocardiography showed sinus rhythm with slight ST-depressions in the leads V4–6.

Bedside echocardiography revealed regional hypokinesia in the lateral area of the left ventricle, and an estimated left ventricular ejection fraction (LVEF) of 20–30%.

The patients’ blood samples demonstrated abnormal with a troponin T of 300 ng/L, white blood cell count of 39 × 10^9^/L and normal c-reactive protein.

**Figure 2:**
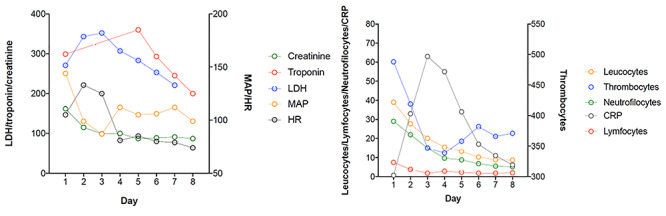
Hemodynamics and biomarkers as measured under admission.

**Figure 1:**
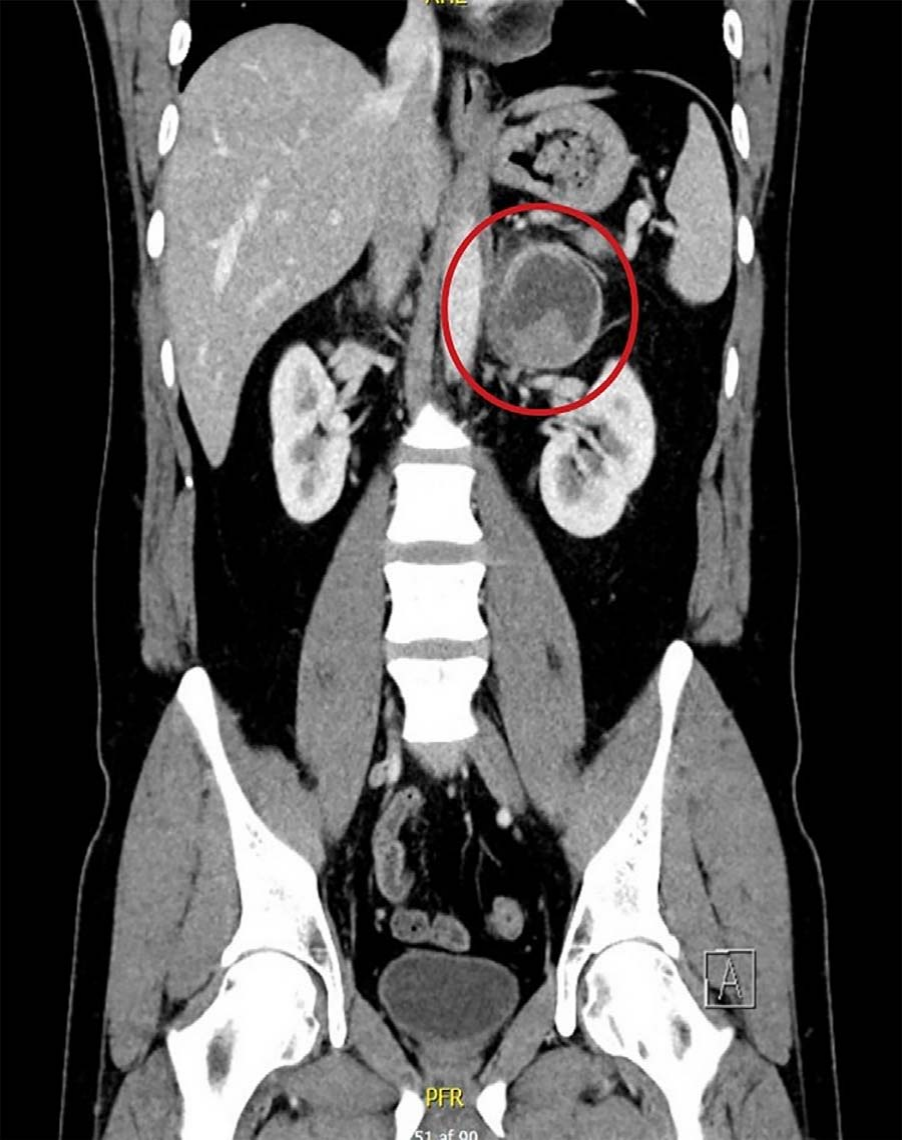
CT scan under admission. Left-sided tumor highlighted inside red circle.

An immediate thoracic/abdominal computed tomography (CT) scan was performed, testing negative for aortic dissection or large pulmonary embolism. However, a 6 cm large tumor in the left adrenal gland was found in Fig. 1.

During the first hours of observation, the patient’s symptoms spontaneously resolved. His vitals normalized and a repeated ABG showed a rising pH (7.33) and decreasing lactate (5.6 mmol/L).

Meanwhile, the patient’s previous history was explored in more detail. The evening before his admission, he had consumed multiple energy drinks, but denies the intake of alcohol or any other substances.

Nine years earlier, he presented with similar symptoms at another hospital, likewise had spontaneously recovered and was quickly discharged without a firm diagnosis. Similarly, 4 years prior to this admission, the patient was acutely hospitalized with headache, back pain, nausea, elevated lactate, white blood cell count and blood pressure. That admission summary described that all symptoms remitted, his vitals as well as blood values normalized over a few hours and the patient was discharged after a day of observation, without finding a plausible explanation to his symptoms.

Due to these findings and the suspicion of pheochromocytoma-induced cardiomyopathy, the patient was admitted.

During the 7-day admission, the patient’s vitals remained normal and blood samples including troponin T slowly normalized. A repeated transthoracic echocardiography showed a LVEF of 45% in Fig. 2.

Plasma cortisol, renin, aldosterone and a 24-hour urine sample for steroid metabolites were within normal range. 3-Metthoxyadrenalin was elevated to 2,5 nmol/L (normal range: < 0,46 nmol/L) as well as 3-Metthoxynoradrenalin at 29,6 nmol/L (normal range < 1,09 nmol/L).

During this observation, the patient reported no symptoms and was discharged for planned surgery in an outpatient setting.

Due to the size of the tumor (6 cm in diameter) and hence a slightly increased risk of malignancy, open left-sided adrenalectomy was performed [1].

The patient was discharged on the fifth postoperative day after uncomplicated surgery and observation period. Follow-up showed normalization of 3-metthoxyadrenalin and 3-metthoxynoradrenalin, and the patient had not experienced any symptoms or complications.

**Figure 3:**
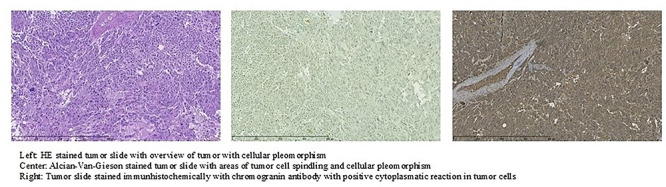
Histology of the patients’ tumor specimen.

The left adrenal gland was found with a well-defined pheochromocytoma with a maximum diameter of 25 mm. The tumor morphology was with the growth of large nests and solid areas, high cellularity and areas with spindle cells as well as areas with profound nuclear pleomorphism and nuclear hyperchromasia. Thus, the Pheochromocytoma of Adrenal Gland Scales (PASS) score was 8 in Fig. 3 [2]. It is not possible to predict the malign potential of a pheochromocytoma from the histopathology, but a PASS score > 4 indicates potential for aggressive clinical behavior. Thus, the patient will be followed with annually blood tests (3-metthoxyadrenalin, 3-metthoxynoradrenalin) lifelong. [2].

## DISCUSSION

Pheochromocytoma is the underlying cause in ~0.2–0.5% of patients presenting with hypertension [3]. The tumor is more commonly seen in people with a positive family history of pheochromocytoma, as well as other genetic syndromes such as MEN-2 and neurofibromatosis [3]. Therefore genetic testing, especially in young patients with pheochromocytoma, should be considered.

Typically, patients presenting with acute adrenergic spells will have at least one or more of the ‘Five P’ symptoms: paroxysmal hypertension, palpitations, perspiration, pallor or pounding headache [1, 3]. However, in the acute setting pheochromocytoma can present with a variety of symptoms mimicking other serious conditions, most commonly acute coronary syndrome.

Cardiomyopathy secondary to pheochromocytoma is a rare complication [4, 5]. Although the conditions pathophysiology can be attributed to the acute secretion of catecholamines, it is debatable whether this fully classifies as cardiomyopathy or should more accurately be defined as catecholamine-induced vasospasm. Since the available literature as well as the majority of case reports describes the condition as catecholamine-induced or takotsubo-like cardiomyopathy, the authors of this article have decided to follow this classification [6–9].

Novel in this case, the patient had presented experienced severe symptomatic episodes several years apart, which all spontaneously resolved within short time. In the years between the episodes, he had been completely asymptomatic. As further mentioned, the night prior to the patients admission, he had been drinking multiple energy drinks over the course of the evening. An admission report from 4 years prior mentions that the patient was working out at a fitness-center, where he experienced sudden onset of his symptoms. Considering this history it seems a possible explanation that the patient’s attacks were triggered by these preceding stressors.

Although a rare condition, pheochromocytoma should be considered as a differential diagnosis, especially in young patients presenting with unexplained hypertension, chest pain and cardiac dysfunction. If a cyclic pattern of symptoms or increased genetic risk can be identified, the relevant examinations should be performed to avoid delayed therapy and further complications of untreated disease.

## DECLARATION

All authors have seen and approved the paper.

The article is the original work of all the authors listed.

The article has not been published elsewhere and is not under consideration elsewhere.
